# Subphenotypes in Non-Syndromic Orofacial Cleft Patients Based on the Tooth Agenesis Code (TAC)

**DOI:** 10.3390/children9030437

**Published:** 2022-03-20

**Authors:** Dimitrios Konstantonis, Maria Nassika, Maria Athanasiou, Heleni Vastardis

**Affiliations:** 1 Department of Orthodontics, School of Dentistry, National and Kapodistrian University of Athens, GR-115 27 Athens, Greece; m.nassika@hotmail.com (M.N.); h.vastardis@gmail.com (H.V.); 2 Athensbestsmiles, In Private Practice of Orthodontics, 49 Alopekis, GR-106 76 Athens, Greece; athanasioumaria994@gmail.com

**Keywords:** cleft lip and palate, non-syndromic orofacial clefts, tooth agenesis, tooth agenesis code (TAC), orofacial cleft subphenotypes

## Abstract

Background: It was the aim of this study to investigate tooth agenesis patterns, which are expressed to different subphenotypes according to the TAC method in a spectrum of non-syndromic orofacial cleft patients. Methods: A total of 183 orofacial cleft patient records were assessed for tooth agenesis and TAC patterns. The association between TAC and sex, and cleft type was examined, and logistic regression models were additionally applied. Additionally, the distribution of missing teeth by cleft type and the tooth agenesis inter-quadrant association were examined. Results: The most frequent cleft type was CLPL (*n* = 72; 39.3%), while the maxillary left lateral incisor was the most frequently missing tooth that was strongly dependent on the cleft type (29.5%, *p* < 0.001). Of the 31 TAC patterns identified, four were the most prevalent and occurred in 80.8% of the sample, while 20 TAC patterns were unique. Cleft type contrary to sex (*p* = 0.405) was found to play a significant role in TAC distribution (*p* = 0.001). The logistic regression’s results suggested that overall, neither sex nor cleft type were associated with tooth agenesis. Prevalence of tooth agenesis in each quadrant clearly depended on cleft type; and there was a strong association found between tooth agenesis in different quadrants. Conclusions: Thirty-one different subphenotypes were identified in TAC patterns. The first four TAC patterns accounted for the 80.8% of the sample’s variability while twenty of the patterns were unique. A strong association was present between TAC pattern and cleft type. No association was found between the sex of the patient, tooth agenesis and TAC patterns. Tooth agenesis depended strongly on the cleft type, and the most frequently missing tooth was the maxillary left lateral incisor. The interquadrant association for tooth agenesis found suggests a genetic link in the etiology of clefts.

## 1. Introduction

Orofacial clefts are considered to be among the most common birth defects, and they require a multidisciplinary treatment approach, which includes surgical correction of the affected area; dental and orthodontic treatment; airway, speech and hearing assessments, as well as psychological sessions throughout childhood and adolescence [1]. Cleft lip and/or palate can occur either in association with other defects (syndromic) or as an isolated trait (non-syndromic) [2]. In a recent systematic review, it has been reported that the prevalence of cleft lip was 0.3 per 1000 live births (95% CI: 0.26−0.34), whereas in the case of cleft lip and palate, it was 0.45 per 1000 live births (95% CI: 0.38−0.52) [3].

The most common dental anomaly found in orofacial cleft patients is tooth agenesis [4,5,6,7]. Still, the most often affected teeth are the lateral incisors at the side of the cleft, followed by the maxillary lateral incisor and the mandibular second premolar in the non-cleft quadrants [5,7,8,9]. Both the prevalence of orofacial clefting as well as tooth agenesis are more frequently observed on the left side, the reason remaining still unknown [8,10,11]. Additionally, it has been reported no significant difference in terms of tooth agenesis is reported between orofacial cleft males and females [4,5,7]. Early craniofacial defects, could lead to jaw abnormalities, and often they are camouflaged by compensatory bone remodeling. Therefore, tooth agenesis and more specifically tooth agenesis patterns may contribute as a biomarker of developmental jaw discrepancies [12].

The genetic link between tooth agenesis and orofacial clefting has been established via the identification of mutations in the MSX1 gene that produce the combined phenotype [13]. Additional genetic variants in MSX1 and PAX9 have been found responsible for clefting and both in situ and ex situ tooth agenesis specifying the topographic variability and underreporting of associated anomalies [14,15]. Genotype–phenotype analyses and WGS technology have revealed that the same causative genes are identified in syndromic and isolated (non-syndromic) cases. Isolated orofacial clefting and tooth agenesis share the same variants in the IRF6 and LRP6 genes and it is a matter of time to decipher the pleiotropic genes involved [16,17].

Identifying certain patterns of tooth agenesis in orofacial non-syndromic cleft patients could result in a better understanding of any genetic background related to specific subphenotypes, hence to an improved link between diagnosis and treatment planning. Using the idea of binary value, van Wick and Tan suggested that “the absence or presence of teeth can be represented by 1 and 0 respectively, and translated into corresponding unique values, the Tooth Agenesis Code (TAC)” [18]. Subsequently, with this method, patterns of tooth agenesis can be assigned unique arithmetical values. The advantage of this scoring system is that each missing tooth has its specific value and the overall score for each quadrant reflects the accurate number and position of tooth agenesis for it. TAC patterns and scores have been proven to be very useful in providing easier data analysis, as they allow for quick recognition of subphenotypes according to specific values [18].

Recently, thirteen different TAC patterns were identified in a sample of non-syndromic patients with bilateral cleft lip and palate and interestingly, the maxillary and/or the mandibular second and/or first premolars were involved in all the patterns [8]. In another study with cleft patients, three different TAC patterns were recognized per quadrant in at least 90% of the sample. Interestingly, symmetrical tooth agenesis patterns were more often observed in the mandible rather than in the maxilla [19]. According to a recent investigation on unilateral and bilateral cleft lip and palate subjects, nineteen different TAC patterns were identified. Additionally, agenesis of the maxillary lateral incisor and of the maxillary second premolar was found in almost all patterns, while the agenesis of the maxillary lateral incisors contributed to the most usual pattern, especially in the maxillary left quadrant [9].

However, there are relatively few reports in the literature that employed the TAC method to assess patterns of tooth agenesis [8,9,19,20]. Additionally, the distribution of TAC patterns in all different types of orofacial clefts and their association with the type of the cleft and patient’s sex has not been thoroughly investigated.

It is therefore the aim of this study to investigate tooth agenesis patterns that are expressed to different subphenotypes according to the TAC method in a spectrum of non-syndromic orofacial cleft patients. Additionally, the association between TAC patterns, sex and cleft type will be examined.

## 2. Materials and Methods

The data for this cross-sectional retrospective study was comprised from consecutive orofacial cleft patient records from the Postgraduate Clinics of the Departments of Orthodontics and Pediatric Dentistry of the Dental School of the National and Kapodistrian University of Athens in Greece. The research protocol was approved by the ethics committee of the University (Ref.312/21.09.2016).

Upon record availability the sample size in a retrospective essay can be often predetermined. An effort was made to collect as many patient records as possible. However, orofacial cleft is one of the rare congenital abnormalities and large data collection is quite difficult to be accomplished in one center. Sample size was assessed using a power analysis which, calculates, for different sample sizes, a probability (power of the analysis), of finding a statistically significant result for a given population. Assuming that the proportion of patients with tooth agenesis is equal to 50%, 166 subjects are required to safeguard that a 99% confidence interval estimation of the pro-portion is within 10% of the true proportion.

Finally, 183 orofacial cleft patients were identified. Of these patients, 115 were male and 68 were female. All patients were born in Greece between 1996 and 2012. The inclusion criteria were:

– Non-syndromic orofacial cleft Caucasian patients;
– Patients older than 8 years old so that agenesis of the second premolars could not be mistakenly documented;
– Complete records including charts; photos; panoramic radiographs; and dental casts;
– No history of prior orthodontic treatment or extraction of permanent teeth;
– No presurgical dentofacial orthopedics, gingivoperiosteoplasty or primary bone grafting so that the tooth agenesis presented could not be regarded as iatrogenic;
– All patient records were taken before the secondary alveolar bone grafting;
– The third molars were not assessed in our investigation.

Since orofacial cleft patients seek dental and orthodontic screening rather early in life, charts and sufficient records including radiographs were available. Specifically, tooth agenesis was identified through patient records, which included intraoral screening; photos, panoramic and when available periapical and cephalometric radiographs. Still, some records included cone-beam computed tomographies (CBCT), and were also taken into consideration. In addition, dental or digital casts were meticulously assessed.

The sample in this investigation was characterized as non-syndromic by the attending medical team. This characterization was based solely on the absence of any other clinical manifestations apart from the orofacial cleft. No DNA analysis was performed in order to exclude the presence of any non-recognizable syndromes.

The principal investigator (D.K.) who initially evaluated the sample for tooth agenesis repeated the measurements once more in order for the intra-observer error to be assessed. Additionally, one of the co-authors (H.V.) evaluated also the whole sample in order to calculate the interobserver error. Kappa statistic was used to calculate the error of the study.

The types of orofacial clefts investigated in our study were: isolated cleft lip at the maxillary right side (CLR); isolated cleft lip at the maxillary left side (CLL); bilateral cleft lip and palate (CLP); unilateral cleft lip and palate at the right side (CLPR); unilateral cleft lip and palate at the left side (CLPL) and isolated cleft palate (CP).

The different patterns of tooth agenesis were identified using the TAC scoring system introduced by Van Wijk et al. [18]. As previously described, using a binary value, the absence or presence of teeth are translated into a specific value within each quadrant. For instance, a patient with agenesis of teeth 22 and 24 will have a score for quadrant 2 (q2) of “010”, which corresponds to the values of the missing teeth of the specific quadrant (a value of 2 for tooth 22 and a value of 8 for tooth 24) and a TAC pattern “000.010.000.000”. Specifying the values of the present and the missing teeth, as can be seen in Table 1, we end up with a specific TAC pattern. In this investigation, we assessed the TAC pattern for each patient.

### Statistical Analysis

Statistical analyses were performed using the R statistical software (4.1.2 (2021-11-01)) [21]. Tooth agenesis was described through the TAC; the percentages of missing teeth in the whole mouth and by quadrant. Fisher’s exact tests were applied to examine the univariable associations between TAC and sex and cleft type and also between tooth agenesis and sex, and cleft type. To adjust for potential confounding effects, multivariable logistic regression models were additionally applied, presenting odds ratios (ORs) for the occurrence of tooth agenesis (at least one tooth missing). Moreover, we evaluated tooth agenesis in each quadrant to determine whether the effects of sex and cleft type are similar for each quadrant. Inter- and intra-examiner agreements were assessed through the Kappa statistic.

## 3. Results

### 3.1. Cleft Distribution and Association with Sex

**Figure 1:**
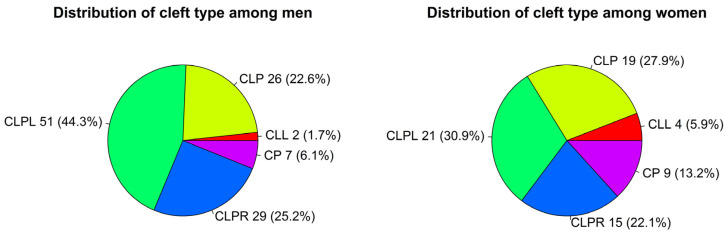
Distribution of cleft type by sex.

In total, 183 cleft patients were examined, of whom, 115 (62.8%) were male and 68 (37.2%) were female. The most frequent cleft type was CLPL (*n* = 72; 39.3%) followed by CLP (*n* = 45; 24.6%); CLPR (*n* = 44; 24.0%); CP (*n* = 16; 8.7%); and CLL (*n* = 6; 3.3%). Men seemed to be more likely to have a CLPL cleft type (44.3%) in comparison to women (30.9%) (Figure 1), although, overall, the association between sex and cleft type did not meet the significance level (*p* = 0.114).

### 3.2. TAC Distribution and Association with Cleft Type and Sex

The distribution of TAC patterns for the whole sample is provided in Table 2. Of all the TAC patterns, four were the most frequently observed and occurred in 80.8% of the sample, while 20 patterns were unique, occurring only once. Restricting to the four most prevalent patterns, the corresponding distribution by sex and cleft type is shown in Table 3. Sex was not found to play a significant role in pattern distribution (*p* = 0.405). The first pattern (000.000.000, p1), which indicated no tooth agenesis, was observed 87 times in all different types of clefts. The second (000.002.000.000, p2) and the third patterns (002.000.000.000, p3) were significantly associated with CLPL and CLPR patients, respectively (*p* = 0.001). The fourth pattern (002.002.000.000, p4) was found to have a significant association with CLP patients (*p* = 0.001).

### 3.3. Tooth Agenesis and Association with Cleft Type and Sex

**Figure 2:**
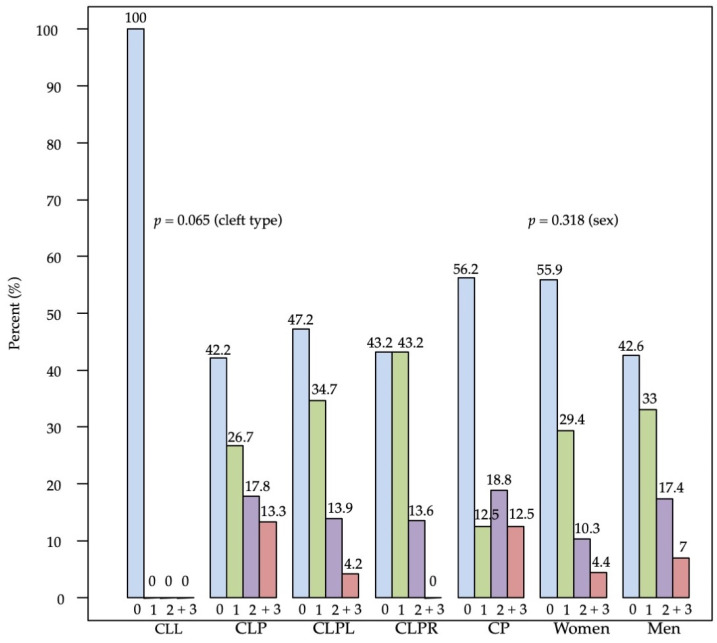
Prevalence of tooth agenesis by cleft type and sex.

The distribution of tooth agenesis by sex and cleft type is presented in Figure 2; overall tooth agenesis was observed in 96 patients (52.5%). Sex was not associated with the prevalence of tooth agenesis (*p* = 0.343), though there was a marginally non-significant result for the relation of cleft type and tooth agenesis (*p* = 0.065). However, this was mainly due to lack of tooth agenesis in CLL patients; excluding such patients, the association diminished (*p* = 0.114) (data not shown).

### 3.4. Inter-Quadrant Association

**Figure 3:**
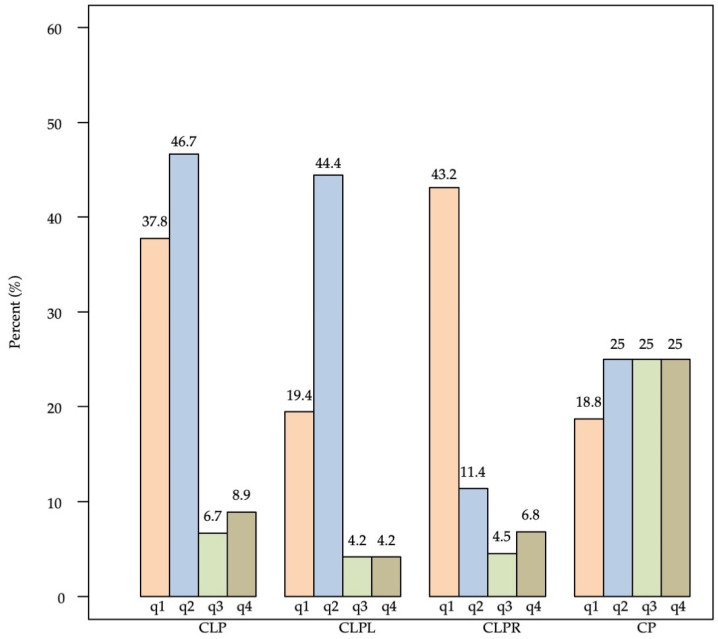
Prevalence (%) of tooth agenesis (at least one tooth being missing) in each quadrant (q1, q2, q3, q4) by cleft type.

Tooth agenesis in the first, second, third, and fourth quadrant occurred in 53 (29%), 62 (33.9%), 12 (6.6%), and 14 (7.7%), (data not shown). Prevalence of tooth agenesis in each quadrant clearly depended on cleft type; e.g., for quadrant 1 (q1), 19.4% for CLPL patients, whereas 43.2% for CLPR patients (Figure 3). There was a strong association between tooth agenesis in different quadrants (Table 4). For example, having tooth agenesis in the q1 significantly increases the likelihood of tooth agenesis in the q2 and q3 (quadrant 3).

### 3.5. Odds Ratios (95% CI and p) for the Association of Tooth Agenesis (at Least One Tooth Being Missing) with Gender and Cleft Type

The results for the logistic regression models are provided in Table 5. CLL type was excluded in the regression analysis due to a very low number of individuals. Overall, neither sex nor cleft type were associated with tooth agenesis (at least one tooth being missing); however, men had 59% (95%CI: from −15% to 199%) higher odds of having tooth agenesis in the whole mouth compared to women, with adjustment for cleft type; however, the association was nonsignificant (*p* = 0.149). CLPL patients have 23% lower odds of having tooth agenesis compared to CLP patients, accounting for sex, but without reaching statistical significance. Cleft type was associated with the presence of tooth agenesis (adjusting for sex) in q1 (*p* = 0.022). For example, CLPL patients have 63% (95% CI: from 13% to 84%) lower odds of having tooth agenesis in q1 compared to CLP patients (*p* = 0.023). For the q2, though, there was not significance difference between CLP and CLPL, whereas CLPR had the lowest probability of missing teeth (*p* < 0.001) (Table 4). For all quadrants, there was no significant association between tooth agenesis and sex adjusted for cleft type.

### 3.6. Most Frequently Missing Teeth by Cleft Type

The number of missing teeth ranged from 1 to 10. The percentages of missing teeth by cleft type are presented in Table 6. When comparing the percentages of missing teeth by cleft type, Bonferroni correction for multiple testing was performed. That is, differences were reported as significant when *p* < (0.05/20) = 0.0025. Overall, 22 was the most frequently missing tooth strongly depending on cleft type (29.5%, *p* < 0.001) followed by 12 (24.0%, *p* = 0.006); 15 (6.6%, *p* = 0.872); 35 (5.5%, *p* = 0.014) and 45 (4.9%, *p* = 0.166).

### 3.7. Error of the Study

All cases were reassessed for tooth agenesis. Then, the error of the study was calculated through the Kappa statistic. Excellent inter- and intra-examiner agreement was found, yielding kappa coefficients from 0.93 to 1.00. In fact, agreement was perfect for all teeth except for one disagreement in one patient for tooth 25.

## 4. Discussion

Most of the studies in the literature either have not distinguished between left and right sides of the clefts or have not included them as separate groups in the statistical analysis. Hence, the reported results regard patients either with unilateral or bilateral cleft lip and palate, the side not being specified. Since the investigation of TAC patterns is a detailed examination of subphenotypes based on tooth agenesis and the side of the dentition plays a major role in the TAC pattern identification and classification, we decided to also consider in our study the side of the cleft in order to investigate any potential associations.

In one of our previous studies, we investigated the prevalence of tooth agenesis and structural tooth discrepancies like tooth malformation and microdontia, in a spectrum of non-syndromic oral cleft patients and their association with the type of the cleft and patient’s sex [5]. Nowadays, recent research trends demand a more thorough and longitudinal phenotyping of the cleft cases in order to elucidate genotype-phenotype associations [22,23]. The TAC patterns and their association with cleft type, sex and tooth agenesis assessed in this investigation can contribute greatly towards this direction.

The patients in this study did not receive any DNA testing to exclude any underlying not recognizable syndrome. However, relevant reports on orofacial clefts suggest the need for early genetic counselling and appropriate follow-ups [15]. Additionally, recent updated guidelines for the comprehensive treatment of oral cleft patients have been suggested [24].

The most frequent cleft type in our sample was CLPL (*n* = 72; 39.3%) in agreement with previous reports [5,8,10,25] followed by CLPR (*n* = 44; 24.04%). Interestingly, CLPL was more frequently observed in men (44.3%) compared to women (30.9%); however, the association between cleft type and sex was not statistically significant and this result is coincident with similar studies [5,26].

In the present study, we did not find a significant level of association between the number of overall tooth agenesis and sex (*p* = 0.343), even though males seemed to have higher chances for tooth agenesis compared to females and this finding is also confirmed by several reports [4,5,9,26]. Interestingly, the results of a study conducted in a Korean population showed a gender-dominant pattern with the maxillary lateral incisors and premolars more frequently missing in males than females in the cleft side, while these teeth were more commonly missing in females in the non-cleft side [27].

In accordance with similar studies the maxillary lateral incisors followed by the maxillary right second premolars and the mandibular left and right second premolars were identified as the most frequently missing teeth [4,5,6,19,20]. An interesting finding concurring with other reports was the agenesis of the right central incisors in two CLP and in two CLPR patients and of the left central incisors in three CLP and one CLPL patients [6,19,20].

The tooth agenesis prevalence in the non-cleft population is significantly smaller than the 52.5% found in our study ranging between 6.4 to 7.1% and depends on the race; the ethnicity and the sex [28,29,30]. A notable remark was made by Lagana that dental agenesis presents often at the distal teeth of each homogenous group of teeth: lateral incisors; second premolars; and third molars [30].

With regard to the association between the cleft type and the agenesis of individual teeth a significant association was found as expected between the CLPL patients and the agenesis of the maxillary left lateral incisor (22) as well between the CLPR patients and the agenesis of the maxillary right lateral incisor (12). This finding supports the hypothesis of the cleft side agenesis of the lateral incisor the clarification of which remains very interesting [7,8,9,31]. Similarly significant and in accordance with previous reports, was the agenesis of mandibular left second premolar (35) observed in the CP patients [5]. It is interesting that in our study the mandibular right second premolar (45) was not linked to any cleft type unlike in other references [7,8,9,19].

A noticeable point is the distribution of tooth agenesis among the four quadrants. Our results showed that tooth agenesis was more likely to occur in the second quadrant (33.9%), followed by the first quadrant (29%). Additionally, tooth agenesis depended clearly on cleft type. Additionally, in the literature, tooth agenesis appeared more frequently in the upper arch [9,19], while a significant association between maxillary tooth agenesis and cleft side was also reported [5,8,20]. Still, concurring with our results various literature reports suggest that the most frequent teeth missing outside the cleft area were the second maxillary and/or mandibular premolars, thus implying a genetic link between tooth agenesis and orofacial clefts [4,26,32].

A significant association was found regarding the possibility of tooth agenesis between q1 and q2. A substantial percentage of the 53 patients (50.9%) who presented with agenesis in q1 presented also with agenesis in q2. Furthermore, the association between q1 and q3 was also significant since 13.2% of the 53 patients with tooth agenesis in q1 presented also with tooth agenesis in q3. The results suggest that a patient with tooth agenesis in q1 is more probable to present also with tooth agenesis in q2 or q3 than a patient without tooth agenesis in q1.

The quadrant associations suggest that tooth agenesis in orofacial clefts is not due to the disrupting process of the cleft itself, which causes a bone defect with accompanying defects, but rather to a genetic link. However, it was recently suggested that the etiology of the orofacial clefts, syndromic or not, is not simply attributed to Mendelian inheritance, but it is also the result of multifactorial causes that encompass polygenic backgrounds and environmental exposures [33,34].

Although TACs are a valuable means of identifying tooth agenesis patterns, only a few studies have implemented them in order to classify dental subphenotypes of non-syndromic orofacial cleft patients. Nevertheless, to our knowledge, there is no other research in the current literature that evaluated tooth agenesis using the TAC method in the five aforementioned different types of orofacial clefts.

In our study, 31 different TAC patterns were identified. The first four TACs accounted for the 80.8% of the sample’s variability characterizing 148 out of 183 patients as follows: no tooth agenesis (000.000.000.000, p1) (47.5%); maxillary left lateral incisor agenesis (000.002.000.000, p2) (16.4%); maxillary right lateral incisor agenesis (002.000.000.000, p3) (9.8%); and maxillary left and right lateral incisor agenesis (002.002.000.000, p4) (7.1%). Furthermore, 20 of the patterns observed were unique thus they were observed only in one patient. Examining the above patterns and considering the number of missing teeth in each pattern, the tooth agenesis presented could be identified as hypodontia, a term that is used if less than six teeth are missing, or oligodontia, a term used for more than six teeth missing. In our sample, only three patterns with oligodontia were identified, suggesting that orofacial cleft patients present mainly with hypodontia.

A recent investigation on bilateral cleft and palate reported 53 different TAC patterns, with 30 of them being unique. The authors reported as most prevalent patterns the four patterns (p1, p2, p3, p4), which were also identified in our study showing mainly either lack of tooth agenesis or maxillary lateral incisor agenesis, being thus in total agreement with our findings [20]. Additionally, in a successive study by the same investigator, unilateral cleft lip and palate patient records were evaluated, and 13 different TAC patterns were identified, with six of them being unique. The two prevalent patterns were those who indicated no tooth agenesis and maxillary right lateral incisor agenesis also found among the four more prevalent patterns in our study (p1 and p3) [8]. Still, in agreement with our findings, the involvement of the cleft side lateral incisor with the non-cleft side lateral incisor and/or the second premolars, in most of the TAC patterns, was reported [8,9].

In our study, there was no significant association neither between TAC patterns and sex nor between tooth agenesis (at least one tooth missing) and sex. Yet, previous similar investigations that evaluated orofacial cleft patients have not examined the aforementioned sex–TAC association. However, concurring results with ours suggest no association between sex and tooth agenesis [8].

Additionally, we found that cleft type, contrary to the lack of association with patient’s sex, associated significantly with the TAC patterns. Specifically, considering the significant association of patterns p1 and p2 to the CLPL and CLPR groups, our findings concur with those of similar studies [8,9,19,20].

In agreement with previous reports, the maxillary lateral incisors and/or the maxillary or mandibular second premolars were the most frequently teeth missing [8,9,19,20]. With regard to the mandibular second premolars, the agenesis of the teeth 35 and 45 was found in 5.5% and 4.9% of our sample, respectively, but unlike the maxillary incisor agenesis, did not contribute to a frequently observed TAC pattern. Nevertheless, TAC patterns with agenesis of the mandibular second premolars were seen immediately after the most frequent TAC patterns, being thus in agreement with previous reports [9,20].

In recent decades, significant progress has been made in decoding the complex genetics of tooth agenesis [35]. The majority of the responsible mutations (91.9) have been identified in only seven genes: PAX9, WNT10A, EDA, MSX1, LRP6, AXIN2x, and WNT10B [23]. Still, tooth development is coordinated by a complex signaling network including the Wnt, Eda/Edar/NF-κB and TGF-β/BMP pathways. Of the above-mentioned genes PAX9 seems to have a key-role in inducing activation of both TGF-β/BMP and Wnt pathways, which are also involved in organogenesis [36]. Recently, it was suggested that both tooth agenesis and craniofacial development share common genetic mechanisms [37].

Currently, the investigation of the prevalence and the distribution of orofacial clefts is considered of paramount importance in elucidating the genetic architecture as well as the pathways that can possibly describe their etiology. Orofacial clefts comprising a spectrum of diverse disorders exhibit a large number of different subphenotypes that range from a single tooth agenesis to a distinct craniofacial morphology [38].

The present investigation provides identification and thorough analysis of different orofacial cleft subphenotypes based on tooth-agenesis patterns that use the TAC methodology. Future studies investigating associations between cleft types, their subphenotypes and genetic variants should be considered. The results of this study empower the clinician with a broader knowledge of the association of tooth agenesis patterns with all types of orofacial clefts in both male and female patients, thus improving diagnosis and appropriate treatment planning.

### Limitations

One of the present study’s limitations was the sample size and the unequal distribution of cleft types, yet orofacial clefts comprise a rare disease and large data collection is rather challenging. The restriction on the Caucasian race constitutes another limitation advising for future investigations to examine different racial groups. Additionally, the sample of this investigation was characterized as non-syndromic on the absence of any other clinical manifestations. No DNA genetic test was performed, and this comprises a limitation.

## 5. Conclusions

A large number of different TAC patterns can be identified in orofacial cleft patients. Many of the TAC patterns are unique, thus occurring only once. The first four TAC patterns accounted for the 80.8% of the sample’s variability and included lack of agenesis followed by unilateral or bilateral agenesis of the maxillary lateral incisor. TAC patterns presented a strong association with cleft type. There was no association found between the sex of the patient, tooth agenesis and TAC patterns. In orofacial cleft patients, the most prevalent cleft type was CLPL followed by CLPR. Half of the orofacial patients presented with tooth agenesis which depended strongly on the cleft type. The most frequently missing tooth was the maxillary left lateral incisor. The interquadrant association found suggests that tooth agenesis in orofacial clefts is not due to the disrupting process of the cleft itself but rather to a genetic link.

## Figures and Tables

**Table 1:** Representation of the human dentition consisting of 32 teeth, divided into 4 quadrants (q1, q2, q3, q4). A: tooth numbering according to the FDI World Dental Federation notation. B: application of binary arithmetic to assign unique values to each tooth agenesis (Van Wijk and Tan, 2006).

	Upper Right Quadrant (q1)							Upper Left Quadrant (q2)						
A	17	16	15	14	13	12	11	21	22	23	24	25	26	27
B	64	32	16	8	4	2	1	1	2	4	8	16	32	64
A	47	46	45	44	43	42	41	31	32	33	34	35	36	37
	Lower Right Quadrant (q4)							Lower Left Quadrant (q3)						

**Table 2:** Distribution of tooth agenesis code (TAC), corresponding missing teeth and number of missing teeth (*N*) in the whole sample.

	Overall (*N* = 183)		
Tooth Agenesis Code (TAC)		Tooth/Teeth Missing	*N*
000.000.000.000	87 (47.5%)	None	0
000.002.000.000	30 (16.4%)	22	1
002.000.000.000	18 (9.8%)	12	1
002.002.000.000	13 (7.1%)	12, 22	2
000.000.016.016	3 (1.6%)	35, 45	2
000.000.000.016	2 (1.1%)	45	1
000.016.000.000	2 (1.1%)	25	1
001.000.000.000	2 (1.1%)	11	1
002.001.000.000	2 (1.1%)	12, 21	2
016.000.000.000	2 (1.1%)	15	1
018.000.000.000	2 (1.1%)	12, 15	2
000.000.000.002	1 (0.5%)	42	1
000.000.008.008	1 (0.5%)	34, 44	2
000.000.016.000	1 (0.5%)	35	1
000.003.000.000	1 (0.5%)	21, 22	2
000.006.000.000	1 (0.5%)	22, 23	2
000.010.000.000	1 (0.5%)	22, 24	2
002.000.016.000	1 (0.5%)	12, 35	2
002.002.000.001	1 (0.5%)	12, 22, 41	3
002.010.000.000	1 (0.5%)	12, 22, 24	3
002.018.000.016	1 (0.5%)	12, 22, 25, 45	4
003.000.000.000	1 (0.5%)	11, 12	2
003.002.000.000	1 (0.5%)	11, 12, 22	3
016.002.000.000	1 (0.5%)	15, 22	2
016.002.016.000	1 (0.5%)	15, 22, 35	3
018.002.016.016	1 (0.5%)	12, 15, 22, 35, 45	5
018.016.016.016	1 (0.5%)	12, 15, 25, 35, 45	5
024.024.000.000	1 (0.5%)	14, 15, 24, 25	4
024.024.008.008	1 (0.5%)	14, 15, 24, 25, 34, 44	6
030.027.016.016	1 (0.5%)	12, 13, 14, 15, 21, 22, 24, 25, 35, 45	10
080.080.082.002	1 (0.5%)	15, 17, 25, 27, 32, 35, 37, 42	8

**Table 3:** Distribution of tooth agenesis code (TAC) by gender and cleft type.

	000.000.000.000 (*N* = 87)	000.002.000.000 (*N* = 30)	002.000.000.000 (*N* = 18)	002.002.000.000 (*N* = 13)	Other (*N* = 35)	*p* Value
Sex						0.405 *
Women	38 (55.9%)	8 (11.8%)	6 (8.8%)	3 (4.4%)	13 (19.1%)	
Men	49 (42.6%)	22 (19.1%)	12 (10.4%)	10 (8.7%)	22 (19.1%)	
Orofacial cleft types						0.001 *
CLL	6 (100.0%)	0 (0.0%)	0 (0.0%)	0 (0.0%)	0 (0.0%)	
CLP	19 (42.2%)	6 (13.3%)	3 (6.7%)	6 (13.3%)	11 (24.4%)	
CLPL	34 (47.2%)	21 (29.2%)	3 (4.2%)	4 (5.6%)	10 (13.9%)	
CLPR	19 (43.2%)	2 (4.5%)	12 (27.3%)	2 (4.5%)	9 (20.5%)	
CP	9 (56.2%)	1 (6.2%)	0 (0.0%)	1 (6.2%)	5 (31.2%)	

* Fisher’s exact test for count data with simulated *p*-value (based on 2000 replicates).

**Table 4:** Tooth agenesis between quadrants: inter-quadrant association.

Q1 and Q2			Q2		*p*-Value
			No	Yes	0.002
	Q1	No	95 (73.1%)	35 (26.9%)	
		Yes	26 (49.1%)	27 (50.9%)	
Q1 and Q3			Q3		
			No	Yes	0.020
	Q1	No	125 (96.2%)	5 (3.8%)	
		Yes	46 (86.8%)	7 (13.2%)	
Q1 and Q4			Q4		
			No	Yes	0.071
	Q1	No	123 (94.6%)	7 (5.4%)	
		Yes	46 (86.8%)	7 (13.2%)	
Q2 and Q3			Q3		
			No	Yes	0.222
	Q2	No	115 (95.0%)	6 (5.0%)	
		Yes	56 (90.3%)	6 (9.7%)	
Q2 and Q4			Q4		
			No	Yes	0.185
	Q2	No	114 (94.2%)	7 (5.8%)	
		Yes	55 (88.7%)	7 (11.3%)	
Q3 and Q4			Q4		
			No	Yes	<0.001
	Q3	No	166 (97.1%)	5 (2.9%)	
		Yes	3 (25.0%)	9 (75.0%)	

**Table 5:** Odds ratios (95% CI and p) for the association of tooth agenesis (at least one tooth being missing) with gender and cleft type. Tooth agenesis was evaluated in the whole mouth and within each quadrant.

Factors	OR	Lower	Upper	*p*-Value
Sex				
Women	Ref			
Men	1.59	0.85	2.99	0.149
Cleft type				0.799
CLP	Ref			
CLPL	0.77	0.35	1.63	0.493
CLPR	0.93	0.39	2.16	0.857
CP	0.6	0.18	1.92	0.392
In the first quadrant				
Sex				
Women	Ref			
Men	1.56	0.77	3.25	0.225
Cleft type				0.022
CLP	Ref			
CLPL	0.37	0.16	0.87	0.023
CLPR	1.21	0.51	2.86	0.661
CP	0.4	0.08	1.48	0.201
In the second quadrant				
Sex				
Women	Ref			
Men	1.68	0.84	3.46	0.147
Cleft type				0.002
CLP	Ref			
CLPL	0.85	0.4	1.83	0.681
CLPR	0.14	0.04	0.39	<0.001
CP	0.4	0.1	1.38	0.167
In the third quadrant				
Sex				
Women	Ref			
Men	0.69	0.2	2.45	0.555
Cleft type				0.082
CLP	Ref			
CLPL	0.64	0.11	3.63	0.597
CLPR	0.69	0.09	4.37	0.69
CP	4.46	0.86	25.55	0.074
In the fourth quadrant				
Sex				
Women	Ref			
Men	1.35	0.42	4.89	0.621
Cleft type				0.078
CLP	Ref			
CLPL	0.43	0.08	2.05	0.285
CLPR	0.73	0.14	3.53	0.696
CP	3.58	0.74	17.62	0.105

**Table 6:** Missing teeth by cleft type in a total of 183 cleft patients (CLL patients are not shown as no agenesis occurred).

	CLP (*N* = 45)	CLPL (*N* = 72)	CLPR (*N* = 44)	CP (*N* = 16)	Total (*N* = 183)	*p* Value
11	2 (4.4%)	0 (0.0%)	2 (4.5%)	0 (0.0%)	4 (2.2%)	0.149
12	15 (33.3%)	10 (13.9%)	17 (38.6%)	2 (12.5%)	44 (24.0%)	0.006
13	1 (2.2%)	0 (0.0%)	0 (0.0%)	0 (0.0%)	1 (0.5%)	0.593
14	2 (4.4%)	1 (1.4%)	0 (0.0%)	0 (0.0%)	3 (1.6%)	0.593
15	4 (8.9%)	5 (6.9%)	2 (4.5%)	1 (6.2%)	12 (6.6%)	0.872
16	0 (0.0%)	0 (0.0%)	0 (0.0%)	0 (0.0%)	0 (0.0%)	
17	0 (0.0%)	0 (0.0%)	0 (0.0%)	1 (6.2%)	1 (0.5%)	0.090
21	3 (6.7%)	1 (1.4%)	0 (0.0%)	0 (0.0%)	4 (2.2%)	0.223
22	17 (37.8%)	30 (41.7%)	4 (9.1%)	3 (18.8%)	54 (29.5%)	<0.001
23	0 (0.0%)	1 (1.4%)	0 (0.0%)	0 (0.0%)	1 (0.5%)	1.000
24	3 (6.7%)	2 (2.8%)	0 (0.0%)	0 (0.0%)	5 (2.7%)	0.236
25	4 (8.9%)	2 (2.8%)	1 (2.3%)	1 (6.2%)	8 (4.4%)	0.353
26	0 (0.0%)	0 (0.0%)	0 (0.0%)	0 (0.0%)	0 (0.0%)	
27	0 (0.0%)	0 (0.0%)	0 (0.0%)	1 (6.2%)	1 (0.5%)	0.090
31	0 (0.0%)	0 (0.0%)	0 (0.0%)	0 (0.0%)	0 (0.0%)	
32	0 (0.0%)	0 (0.0%)	0 (0.0%)	1 (6.2%)	1 (0.5%)	0.090
33	0 (0.0%)	0 (0.0%)	0 (0.0%)	0 (0.0%)	0 (0.0%)	
34	0 (0.0%)	1 (1.4%)	1 (2.3%)	0 (0.0%)	2 (1.1%)	0.792
35	3 (6.7%)	2 (2.8%)	1 (2.3%)	4 (25.0%)	10 (5.5%)	0.014
37	0 (0.0%)	0 (0.0%)	0 (0.0%)	1 (6.2%)	1 (0.5%)	0.090
41	0 (0.0%)	0 (0.0%)	0 (0.0%)	1 (6.2%)	1 (0.5%)	0.090
42	0 (0.0%)	0 (0.0%)	1 (2.3%)	1 (6.2%)	2 (1.1%)	0.053
43	0 (0.0%)	0 (0.0%)	0 (0.0%)	0 (0.0%)	0 (0.0%)	
44	0 (0.0%)	1 (1.4%)	1 (2.3%)	0 (0.0%)	2 (1.1%)	0.792
45	4 (8.9%)	2 (2.8%)	1 (2.3%)	2 (12.5%)	9 (4.9%)	0.166
47	0 (0.0%)	0 (0.0%)	0 (0.0%)	0 (0.0%)	0 (0.0%)	

## Data Availability

The data presented in this study are available on request from the corresponding author. The data are not publicly available due to GDPR policy.
